# Atomic replacement effects on the band structure of doped perovskite thin films

**DOI:** 10.1038/s41598-019-44104-7

**Published:** 2019-05-24

**Authors:** S. L. Cheng, C. H. Du, T. H. Chuang, J. G. Lin

**Affiliations:** 1 Center for Condensed Matter Sciences, National Taiwan University, Taipei, 10617 Taiwan; 2 Department of Physics, Tamkang University, Tamsui, 251 Taiwan; 3 Department of Materials Science and Engineering, National Taiwan University, Taipei, 10617 Taiwan; 4 Center for Atomic Initials for New Materials, National Taiwan University, Taipei, 10617 Taiwan

**Keywords:** Materials science, Surfaces, interfaces and thin films

## Abstract

The potential applications of perovskite manganite R_1-x_A_x_MnO_3_ (R = rare earth element; A = Sr, Ca) thin films have been continuously explored due to their multi-functional properties. In particular, the optimally hole-doped La_0.67_Ca_0.33_MnO_3_ thin film demonstrates a colossal magneto-resistance that is beneficial to the performance of spintronic devices. To understand the effect of R and A ions on the material properties, we systematically measure the resistivity, magnetization, and electronic energy states for three optimally hole-doped R_0.67_A_0.33_MnO_3_ thin films with R = La, Sm and A = Sr, Ca. Various energy parameters are derived based on the X-ray absorption and X-ray photoelectron spectra, including the band gap, the charge frustration energy and the magnetic exchange energy. It is interesting to find that the replacement of La with Sm is more effective than that of Sr with Ca in terms of tuning the electrical property, the Curie temperature, and the band gap. The strain-induced reduction of the O 2*p*- Mn 3*d* hybridization and the interplay of R/A site disorder and strain effect are discussed. The results of this study provide useful information for the band design of perovskite oxide films.

## Introduction

Perovskite manganite R_1-x_A_x_MnO_3_ (R = rare earth element; A = alkaline metal) has attracted long standing attention because of its fascinating properties related to the correlations between spin, charge, and orbital degrees of freedom. In 1994, the discovery of colossal magneto-resistance in ferromagnetic La_0.67_Ca_0.33_MnO_3_ thin films activated the potential applications of perovskite oxides on magnetic recording media^1^. However, there are still many issues to resolve before the practical integration of perovskite oxides into semiconductor devices. They include developing an understanding of the band structure and controlling the band gap. Similar to conventional semiconductors, the electronic structure of R_1-x_A_x_MnO_3_ can be tuned using either hole doping (x > 0)^2–8^ or internal strain^7,8^. Unlike conventional semiconductors, the transport mechanism of R_1-x_A_x_MnO_3_ involves strong electron-electron correlations, leading to complex transport properties different from the typical metallic or insulating behavior. Strong on-site repulsion (Hubbard U-term) between 3*d* electrons may cause an integer-filled 3*d*^n^ configuration, resulting in a Mott-insulator. Before turning into a Mott-insulator, the degree of hybridization of O - 2*p* and Mn - 3*d* bands may be modified to cover a wide range of transport properties from the charge-transfer type (such as AMnO_3_) to Mott-Hubbard type (such as in RMnO_3_)^9–12^. Based on the Mott-Hubbard type theory, the charge fluctuations of between *d*^n^(*i*)*d*^n^(*j*) and *d*^n-1^(*i*)*d*^n+1^(*j*) states are strongly suppressed by high exchange energies, with *i* and *j* being different transition-metal sites. Additionally, the unique characteristics of the intermedium state open a new channel to tune the gap energy via ionic valence. Many reports suggest that the magnetic and electrical properties of mixed-valence R_1-x_A_x_MnO_3_ compounds could be modified either by changing the doping level, x, or by inserting various rare earth elements^13–17^. Accordingly, a reduction of the effective ionic size r_eff_ [r_eff_ = (1 − x)r_Rare_ + xr_Alkai_] would enhance the local deformation of MnO_6_ octahedron and narrow the effective width of the e_g_ band. An increment of the ionic radii mismatch between R and A could also induce quenched lattice disorder^16^, which influences the stability of the ferromagnetic/orbital phase. Here, the x-effect is more complex since the variation of x could simultaneously change both the carrier concentration and the effective ionic size. Therefore, a simple approach to study the pure ionic effect is to fix the doping concentration. To design a functional read-write device, the required energies of switching between different electronic states are crucial. Overall, the correlation between crystal structure, electronic energy state, the electrical transport and magnetic property in manganite oxides is not yet fully understood^14–17^.

Although many papers report the magnetic and electronic properties of R_1-x_A_x_MnO_3_^18–20^, they mostly report on bulks and much less often on films^21–24^. For future applications of manganite films, a detailed characterization of the film properties is required. Particularly for the narrow bandwidth manganites with R = Pr, Sm and Nd, the competing magnetic and electronic states often reside in close energetic proximity, and therefore their physical properties are sensitive to atomic replacement^24^. In thin film form, the interfacial strain could play an important role in addition to the atomic replacement. In this work, x is fixed as 0.33 to study the effects of R/A replacement on the properties of R_1-x_A_x_MnO_3_ thin films. We report a systematic analysis on the electronic structures of R_0.67_A_0.33_MnO_3_ (R = La, Sm; A = Sr, Ca) thin films with X-ray absorption spectroscopy (XAS) and X-ray photoelectron spectroscopy (XPS). Using La_0.67_Sr_0.33_MnO_3_ (LSMO) as a reference compound, we find that replacing a La-ion with a Sm-ion is more effective than replacing Sr with Ca to modify the band structure, regardless of the similar effective ionic sizes in La_0.67_Ca_0.33_MnO_3_ (LCMO) and Sm_0.67_Sr_0.33_MnO_3_ (SSMO). This phenomenon has been observed in bulk samples and explained by the quenched disorder^16^, but it could be more (or less) profound in thin film form due to the additional interfacial strain. To our knowledge, this article is the first report discussing the strain effect on the electronic structure of SSMO thin film. The mixed phase of ferromagnetic and antiferromagnetic states makes the properties of SSMO more sensitive to changes in crystal structure^20^. The significant difference of physical properties between bulk and thin film has important implications for device design.

## Results

**Figure 1:**
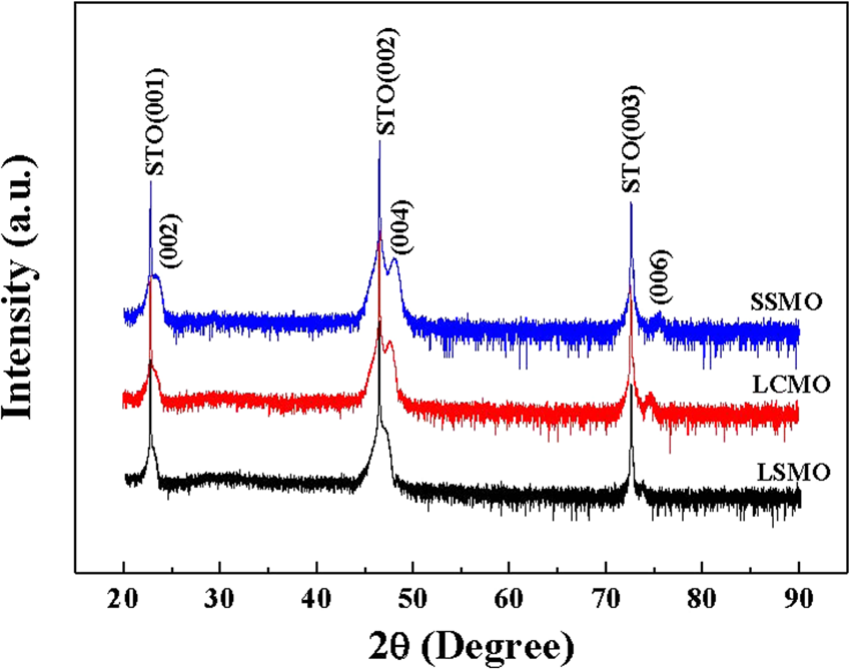
θ−2θ XRD diffraction patterns for LSMO, LCMO, and SSMO films, following the sequence from bottom to top.

θ-2θ XRD patterns of LSMO, LCMO, and SSMO thin films of 10 nm thickness are plotted in Fig. 1. There is no trace of any impurity peaks in XRD patterns. Only the (00 *l*) peaks of STO and the films are observed, indicating that all films are *c*-oriented along the surface-normal direction. Lattice parameters of films are calculated based on the (00 *l*) diffraction peaks. The obtained *c*-lattice parameter of orthorhombic structure is 7.69 Å for LSMO, 7.63 Å for LCMO, and 7.56 Å for SSMO films (all shorter than their bulk values), suggesting a tensile interfacial strain on the planes of all three films. However, the reciprocal space mapping data of XRD is desirable, which could provide information on in-plane lattice constants.

**Figure 2:**
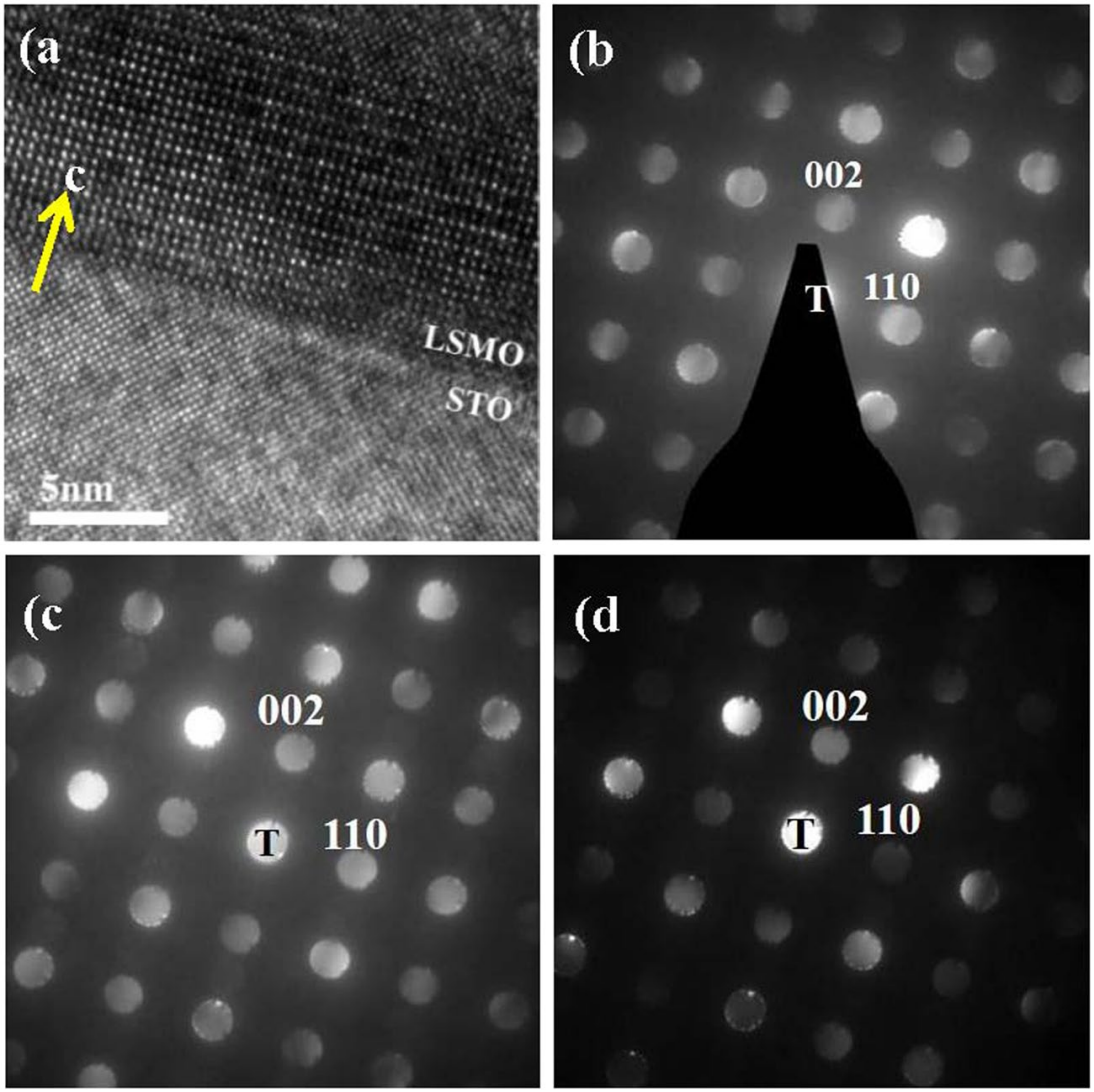
(**a**) Cross-sectional HRTEM image of (001) STO/LSMO. The nano-beam diffraction patterns for (**b**) LSMO, (**c**) LCMO and (**d**) SSMO.

A cross sectional FETEM image for STO/LSMO single layer is shown in Fig. 2(a). A smooth interface between the substrate and the film is demonstrated with a clear image of long-range ordered atoms in the LSMO film as well as in those of LCMO and SSMO. The arrow points to the direction of surface-normal films. Figures 2(b–d) are the patterns of nano-beam electron diffraction (NBD) for LSMO, LCMO, and SSMO films, respectively. d_002_ and d_110_ mark the distance between the incident beam **T** and diffraction spots of (002) and (110), respectively. The ratio of d_002_ and d_110_ is estimated to be about 1, consistent with the database of orthorhombic structures with the space group of Pnma^25–27^.

**Figure 3:**
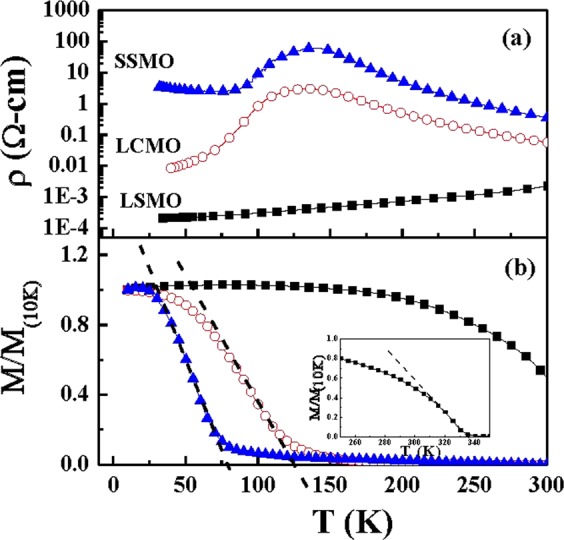
ρ(T) curves (**a**) and the normalized M(T) curves (**b**) for LSMO (■), LCMO (○), and SSMO (▲) film. The inset of (**b**) is the M(T) curve of LSMO film in the range of 250 K~350 K.

Curves of resistivity (ρ) vs. temperature (T) from 300 to 30 K for three films are plotted in Fig. 3(a). An insulator-to-metal transition temperature (T_IM_) is defined at the position of the peak (135 K) for LCMO and SSMO films, while the metal-like behavior of LSMO film indicates its T_IM_ is higher than 300 K. The value of ρ at 300 K for SSMO film is 355 mΩ-cm, which is 150 times larger than that of LSMO (2.3 mΩ-cm) and 6 times larger than LCMO (56 mΩ-cm). The T-dependent normalized magnetization M/M(10 K) from 300 to 10 K is plotted in Fig. 3(b). The paramagnetic-ferromagnetic transition temperature T_c_ is defined as the crossing point of an extracted line of the paramagnetic state and the *x*-axis in the curves of M(T) as indicated with dash lines; it is determined as 331 K for LSMO (see the inset), 126 K for LCMO, and 79 K for SSMO. The values of T_IM_ and T_c_ are very close for LSMO but different by 56 K for SSMO, indicating the double-exchange model may not be applicable in SSMO. In addition, the magnetic moments obtained at 10 K for these three films are very different. It is 2.7 μ_B_/Mn for LSMO, 1.2 μ_B_/Mn for LCMO, and 0.32 μ_B_/Mn for SSMO.

**Figure 4:**
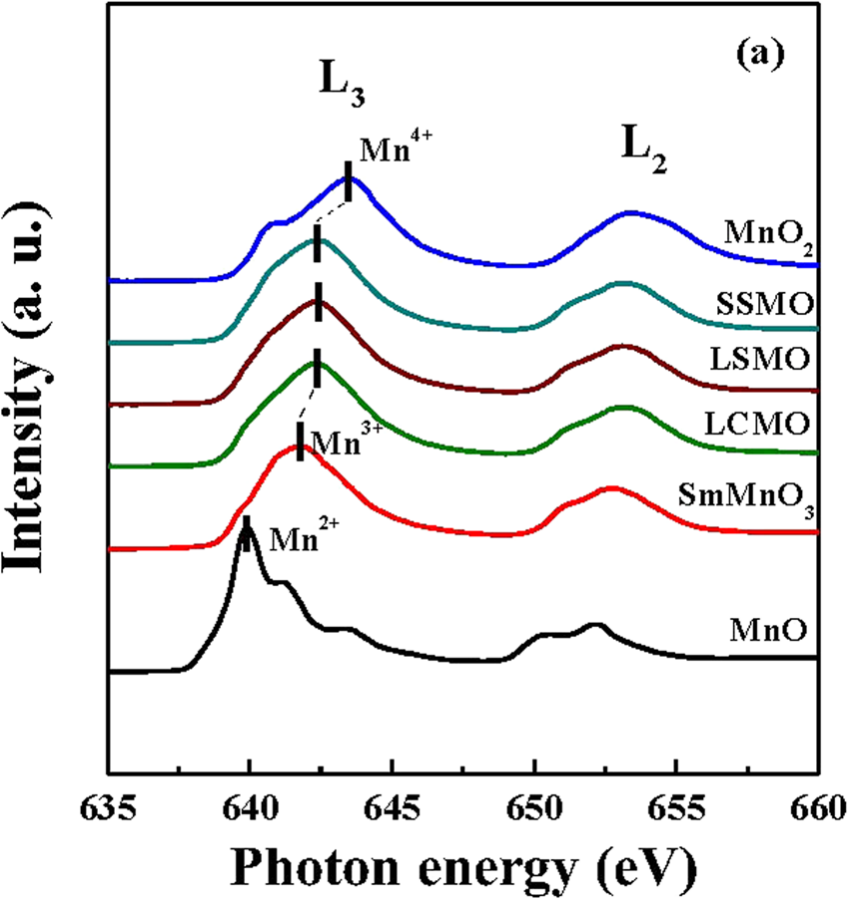
Mn L_2,3_-edge absorption spectra for the polycrystalline samples of MnO (black line), SmMnO_3_ (red line) and MnO_2_ (blue line), and for the film samples of LCMO (green line), LSMO (brown line), and SSMO (cyan line).

Figure 4 shows the absorption spectra of Mn 2*p* for all three films, along with those for bulk MnO, SmMnO_3_, and MnO_2_ as the reference spectra of Mn^2+^, Mn^3+^, and Mn^4+^. Accordingly, the energies of the L_3_-edge are 639.9 eV for Mn^2+^, 641.8 eV for Mn^3+^, and 643.5 eV for Mn^4+^. The peak positions of all three films are located at around 642 eV, which is between the energies of Mn^3+^ and Mn^4+^. Thus, the results of absorption spectra confirm a constant mixed-valence of the Mn ion for all three films.

**Figure 5:**
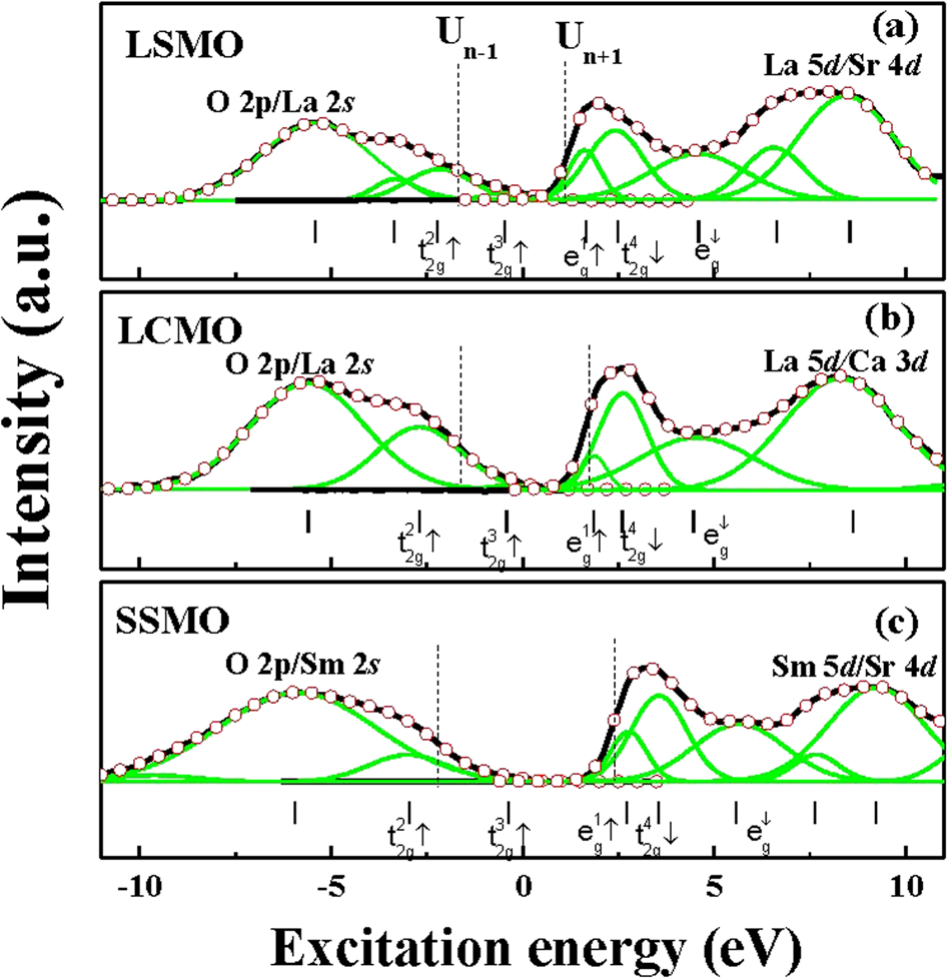
XPS (left-hand side from zero) and XAS spectra (right-hand side from zero) of (**a**) LSMO, (**b**) LCMO and (**c**) SSMO with respect to the patterns from top to bottom. The simulated spectra are drawn by black lines and the observed data are represented by open circle “○”. The simulated spectra are fitted by Gauss functions and displayed as several features drawn by green lines. The five t_2g_ and e_g_ states of Mn 3*d* electrons are indicated at the bottom. The vertical lines at the bottom are the reference energy states obtained from ref. ^25^.

The XPS and XAS data are combined to display the whole range of electronic structure from −11 to 11 eV, as shown in Fig. 5. We set the fermi energy to zero; therefore, negative energies fall in the valence band and positive energies in the conduction band. The zero energy is calibrated with the carbon element and the MnO on the film’s surface for XPS and XAS, respectively. Both the XPS and XAS spectra are fitted with multiple Gaussian functions to determine the energy level of each band. The spectrum within the range of −3 eV and 6 eV is attributed to the hybridization of O 2*p* and Mn 3*d*-electrons, in which the energy structures can be decomposed into five orbital states, ${t}_{2g}^{2}(\,\uparrow \,),\,\,{t}_{2g}^{3}(\,\uparrow \,),\,{e}_{g}^{1}(\,\uparrow \,),\,{t}_{2g}^{4}(\,\downarrow \,)$ and e_g_(↓), plotted as green curves in Fig. 5. The energy levels of (Pr,Nd)_0.7_(Sr,Ca)_0.3_MnO_3_ films are used as references^28^ for the band assignment and marked as vertical lines at the bottom of Fig. 5.

Apart from the orbital energy levels, three energies are extracted as the band gap (E_g_), charge transfer energy E_cf_, and the magnetic exchange energy (E_ex_). E_g_ mimics the band gap in semiconductors and is determined by the energy separation between the two midpoints of the pre-edge feature near E_F_, marked with two dashed lines as U_n+1_ and U_n-1_ in Fig. 5(a–c). The value of E_g_ is estimated at 2.64 eV for LSMO, 3.03 eV for LCMO, and 4.36 eV for SSMO. E_cf_ is determined by the energy separation between ${e}_{g}^{1}(\,\uparrow \,)$ and $\,{t}_{2g}^{3}$(↑) states and estimated at 2.15 eV, 2.25 eV, and 3.09 eV for LSMO, LCMO, and SSMO, respectively. E_ex_ is determined by the energy difference between ${t}_{2g}^{4}(\,\downarrow \,)$ and $\,{t}_{2g}^{3}(\,\uparrow \,)$, which is 2.96 eV, 3.01 eV, and 3.91 eV for LSMO, LCMO, and SSMO, respectively.

## Discussion

From the XPS and XAS data, we conclude that the band structures of LSMO, LCMO, and SSMO are different due to the modification of the hybridization between Mn 3*d* and O 2*p* states. The obtained three energies E_g_, E_cf_, and E_ex_, as well as the orbital energy levels, are listed in Table 1 for LSMO, LCMO, and SSMO films. The energy levels of un-doped bulk samples of LaMnO_3_ and CaMnO_3_ are also listed in Table 1 for reference^29–34^. Compared with LSMO, significant increases in E_g_, E_cf_, and E_ex_ is observed in SSMO, in contrast with a minor change in LCMO. It is interesting to note that the band gaps of LSMO and LCMO differ by 15% with a change of r_eff_ by 0.04 **Å**, while the band gap in SSMO is larger than that of LSMO by 65% with a change of r_eff_ by 0.05 **Å**. This implies that the value of r_eff_ may not be the prime factor influencing the degree of hybridization between O 2*p* and Mn 3*d* band in these films. The distinction between SSMO and the other two samples is reflected from the ρ(T) behaviors of three films. As seen from Fig. 3(a), the ρ-value of SSMO below the transition temperature increases with decreasing temperature, suggesting the ground state of SSMO is an insulating state. Conversely, the ρ-value of LSMO and LCMO below the transition temperature reduces with decreasing temperature, consistent with the nature of metallic state. In addition, the values of T_IM_ (insulator-metal transition) and T_c_ (magnetic transition) deviate by 50 K in SSMO film, while these two values are very close in LSMO and LCMO films.

**Table 1:** Various physical parameters including the effective radius of A-ions r_A_, orbital states $({t}_{2g}^{2},\,{t}_{2g}^{3},\,{e}_{g}^{1},\,{t}_{2g}^{4},\,{e}_{g}^{2},\,and\,{e}_{g}^{3})$, band gap (E_g_), exchange energy (E_ex_), and charge fluctuation energy (E_cf_) for LSMO, LCMO, and SSMO films with the parent compounds LaMnO_3_ and CaMnO_3_ listed as references.

	LSMO	LCMO	SSMO	LaMnO_3_ (refs ^29–32^)	CaMnO_3_ (refs ^32–34^)
*c* (Å)	7.69	7.63	7.56	7.69	7.45
r_A_ (Å)	1.24	1.20	1.19	1.21	1.18
*t*	0.92(9)	0.91(6)	0.91(2)	0.90(4)	0.94(5)
${{\boldsymbol{t}}}_{2{\boldsymbol{g}}}^{2}$ ↑	−2.22	−2.70	−2.96	−1.25	−3.0
${{\boldsymbol{t}}}_{2{\boldsymbol{g}}}^{3}$ ↑	−0.48	−0.41	−0.37	—	—
${{\boldsymbol{e}}}_{{\boldsymbol{g}}}^{1}$ ↑	1.67	1.84	2.72	—	0.2
${{\boldsymbol{t}}}_{2{\boldsymbol{g}}}^{4}$ ↓	2.48	2.6	3.54	—	1.0
${{\boldsymbol{e}}}_{{\boldsymbol{g}}}^{2}$ ↑	—	—	—	2.15	—
${{\boldsymbol{e}}}_{{\boldsymbol{g}}}^{3}$ ↓	4.59	4.46	5.58	—	—
E_g_ (eV)	2.6(4)	3.0(3)	4.3(6)	1.7	1.55
E_ex_ (eV)	2.9(6)	3.0(1)	3.9(1)	3.5	3.0~3.5
E_cf_ (eV)	2.1(5)	2.2(5)	3.0(9)	3.4 ± 0.4	3.2 ± 0.4
Δ(eV)	U > 2.9 eV, and Δ ≤ 2.2 eV^12^.			4.5 ± 0.5	3.0 ± 0.5
U(eV)				3.5 ± 0.3	5.2 ± 0.3

Does the unusual behavior of SSMO film relate to its interfacial strain effect as reflected by the change of c-lattice parameter? It is noted that the c-lattice parameter is also sensitive to the chemical composition, e.g. oxygen or cation deficiency. To avoid an oxygen deficiency, the film was post-annealed in an oxygen atmosphere after growth (as described in the following section). To understand the chemical composition of oxide film, we previously performed the atomic-scale interfacial studies on our Nd_0.35_Sr_0.65_MnO_3_ films and the results proved a well-defined interface with a correct cation composition^35,36^. Therefore, we attribute the changes of c-lattice parameter to the interfacial strain. The interfacial strain ε of SSMO film is compared to those of LSMO and LCMO films. Theoretically, the ε value [defined as (√2 * *a*_STO_ − *a*_bulk_)/*a*_bulk_] between STO substrate (*a* = *b* = *c* = 3.906 Å) and LSMO bulk (pseudocubic, *a* = *b* = 5.472 Å) is 0.95% with respect to both the *a-* and *b*-axes. For LCMO (*a* = 5.460 Å and *b* = 5.476 Å) on STO, ε is around 1.17% along the *a*-axis and 0.88% along the *b*-axis. For SSMO (*a* = 5.418 Å and *b* = 5.430 Å) on STO, ε is 1.95% along the *a-*axis and 1.73% along the *b*-axes. To separate the strain effect from the size effect, we have measured the values of T_C_ and T_IM_ for SSMO bulk, (the data are not shown here). Compared with the properties of SSMO bulk, the difference between T_c_ and T_IM_ of SSMO films (79 K and 136 K, respectively) indeed are much higher than those of the bulk (85 K and 67 K, respectively). It is evident that the tensile strain has a strong influence on the electrical transition but less on the magnetic transition of SSMO films.

According to a previous report on the strain effects of La_1-x_Ba_x_MnO_3_ thin films^22^, the strain effect on the Curie temperature (T_c_) was depending on x. The interfacial compressive strain reduced T_c_ for x = 0.3 and 0.33. This phenomenon was attributed to the competition of strain-induced modification of Mn-O bond length and e_g_ orbital stability. In Eu_0.7_Sr_0.3_MnO_3_ thin films^23^, both the tensile and the compressive strain reduced T_c_, which was correlated to the decrease of saturation magnetization. In contrary, our result of strain effect on T_c_ for SSMO film is different from those of La_0.67_Ba_0.33_MnO_3_ and Eu_0.7_Sr_0.3_MnO_3_. The tensile strain reduces the effective magnetic moment by one order of magnitude but the T_c_ only by 6 K. And T_MI_ is greatly enhanced by near 70 K in SSMO film in comparison with bulk. Considering that a strong A-site disorder in Sm_0.7_Ba_0.3_MnO_3_ bulk could induce a huge separation of T_MI_ and T_c_^16^, it is possible that the anisotropic tensile strain enhances the A-site disorder in SSMO and thus raises T_MI_. Since the magnetic transition is dominated by the major phase of ferromagnetic domains, it is not affected by strain as much as the electrical and electronic properties are.

In summary, the electrical, magnetic, and electronic properties of three optimally hole-doped Perovskite manganite films are systematically investigated. The effects of interfacial strain are compared with that of atomic replacement. The temperature-dependent resistivity data show that the ground state of SSMO film is insulating while it is metallic for LSMO and LCMO films, suggesting the transport mechanism in SSMO film is different from other two samples. In addition, the strain effect on the insulator-metal temperature of SSMO film is much more significant compared with that of LSMO and LCMO films. The energy levels of ${t}_{2g}^{2}(\,\uparrow \,),\,{t}_{2g}^{3}(\,\uparrow \,),\,{e}_{g}^{1}(\,\uparrow \,),{t}_{2g}^{4}(\,\downarrow \,),$ and *e*_*g*_(↓) are identified within the range of the hybridization band. Accordingly, the values of the band gap, charge fluctuation energy, and magnetic exchange energy (E_g_, E_cf_, E_ex_) are obtained as (2.6 eV, 2.1 eV, 2.9 eV) for LSMO film, (3.0 eV, 2.2 eV, 3.0 eV) for LCMO film, and (4.3 eV, 3.0 eV, 3.9 eV) for SSMO film. A large band gap of 4.3 eV in SSMO film is obtained, which may be beneficial to the wide band gap applications.

## Materials and Methods

La_0.67_Sr_0.33_MnO_3_ (LSMO), La_0.67_Ca_0.33_MnO_3_ (LCMO), and Sm_0.67_Sr_0.33_MnO_3_ (SSMO) thin films with a fixed thickness of 10 nm were synthesized and denoted as LSMO, LCMO, and SSMO, respectively. Three films were deposited on a (001) SrTiO_3_ (STO) single crystal using a 248 nm KrF excimer pulsed laser system, at 800 °C with an oxygen pressure of 100 mtorr. After as-grown, the samples were cooled down to 400 °C and *in-situ* annealed in 760 Torr oxygen atmosphere for 1 hour, then again cooled to room temperature. The growth rate is 0.04 Å/sec, allowing one to control the thickness of each film at the nanometer level. The crystalline structure was identified at room temperature with a Cu Kα_1_ (λ = 1.5406 Å) monochromatic x-ray diffraction (XRD) system. The microstructure and interface property were examined using the Field Emission Transmission Electron Microscope (FETEM, FEI Tecnai G2 F20). The temperature (T) dependence of resistivity (ρ) was measured using a standard four-probe method in a closed-cycle refrigerator with an input current ~ 10 μA. Keithley 220 and 182 were used as the current source and voltage meter, respectively. Magnetization (M) as a function of T was obtained with SQUID-VSM instrument (Quantum Design) under the condition of field cooling to 10 K at an in-plane external magnetic field of 100 Oe. The electronic structures were extracted from the XAS and XPS spectra at room temperature. A MnO polycrystalline sample and the carbon element on the film’s surface were referred as an energy calibration standard for absorption and photoelectron spectrum, respectively. The XAS measurements at the O K-edge and Mn L-edge were performed at the beamline 20A1 of the National Synchrotron Radiation Research Center in Hsinchu, Taiwan. The energy resolution was about 0.08 eV at O K-edges and 0.11 eV at Mn L_2,3_-edge, recorded in total electron yield. The slit widths were 20 μm by 20 μm. The XPS measurements were carried out using a spectrometer equipped with a Al Kα source (*hv* = 1486.6 eV) and a standard energy analyzer.
